# Clinical and epidemiologic principles of anthrax.

**DOI:** 10.3201/eid0504.990418

**Published:** 1999

**Authors:** T J Cieslak, E M Eitzen

**Affiliations:** U.S. Army Medical Research Institute of Infectious Diseases, Ft. Detrick, Maryland 21702, USA. Ted_Cieslak@Dtrick.Army.Mil


### Clinical and Epidemiologic Principles of Anthrax

**Theodore J. Cieslak and Edward M. Eitzen, Jr.**
U.S. Army Medical Research Institute of Infectious Diseases,
Ft. Detrick, Maryland, USA

### Background and Epidemiology

Anthrax is one of the great infectious diseases of antiquity. The fifth and sixth plagues in the Bible's book of Exodus (1) may have been outbreaks of anthrax in cattle and humans, respectively. The "Black Bane," a disease that swept through Europe in the 1600s causing large numbers of human and animal deaths, was likely anthrax. In 1876, anthrax became the first disease to fulfill Koch's postulates (i.e., the first disease for which a microbial etiology was firmly established), and 5 years later, in 1881, the first bacterial disease for which immunization was available (2). Large anthrax outbreaks in humans have occurred throughout the modern era—more than 6,000 (mostly cutaneous) cases occurred in Zimbabwe between October 1979 and March 1980 (3), and 25 cutaneous cases occurred in Paraguay in 1987 after the slaughter of a single infected cow (4).

Anthrax, in the minds of most military and counterterrorism planners, represents the single greatest biological warfare threat. A World Health Organization report estimated that 3 days after the release of 50 kg of anthrax spores along a 2-km line upwind of a city of 500,000 population, 125,000 infections would occur, producing 95,000 deaths (5). This number represents far more deaths than predicted in any other scenario of agent release. Moreover, it has been estimated (6) that an aerial spray of anthrax along a 100-km line under ideal meteorologic conditions could produce 50% lethality rates as far as 160 km downwind. Finally, the United States chose to include anthrax in the now-defunct offensive biological weapons program of the 1950s, and the Soviet Union and Iraq also admitted to possessing anthrax weapons. An accident at a Soviet military compound in Sverdlovsk in 1979 resulted in at least 66 deaths due to inhalational anthrax, an inadvertent demonstration of the viability of this weapon. The epidemiology of this inadvertent release was unusual and unexpected. None of the persons affected were children (7). Whether this is due to differences in susceptibility between children and adults or purely to epidemiologic factors (children may not have been outdoors at the time of release) is unclear.

Anthrax is caused by infection with *Bacillus anthracis*, a gram-positive spore-forming rod. The spore form of this organism can survive in the environment for many decades. Certain environmental conditions appear to produce "anthrax zones," areas wherein the soil is heavily contaminated with anthrax spores. Such conditions include soil rich in organic matter (pH $<6.0$) and dramatic changes in climate, such as abundant rainfall following a prolonged drought. Partly because of its persistence in soil, anthrax is a rather important veterinary disease, especially of domestic herbivores. In addition to encountering anthrax while grazing in areas of high soil contamination, these herbivores may also acquire the disease from the bite of certain flies (8). Vultures may mechanically spread the organism in the environment (9). Anthrax zones in the United States closely parallel the cattle drive trails of the 1800s (10).

Anthrax spores lend themselves well to aerosolization and resist environmental degradation. Moreover, these spores, at 2-6 microns in diameter, are the ideal size for impinging on human lower respiratory mucosa, optimizing the chance for infection. It is the manufacture and delivery of anthrax spores in this particular size

Address for correspondence: Theodore J. Cieslak, Operational Medicine Division, USAMRIID, 1425 Porter Street, Ft. Detrick, MD 21702, USA; fax: 301-619-2312, e-mail: Ted_Cieslak@Detrick.Army.Mil.

range (avoiding clumping in larger particles) that presents a substantial challenge to the terrorist attempting to use the agent as a weapon. The milling process imparts a static charge to small anthrax particles, making them more difficult to work with and, perhaps, enabling them to bind to soil particles (11). This, in part, may account for the relatively low secondary aerosolization potential of anthrax, as released spores bind to soil, now clumping in particles substantially in excess of 6 microns. This clumping tendency, together with a high estimated $ID_{50}$ of 8,000-10,000 spores may help explain the rarity of human anthrax in most of the Western world, even in areas of high soil contamination. Other potential bioweapons, such as Q fever and tularemia, have $ID_{50}$ values as low as 1 and 10 organisms, respectively.

### The Disease

Most endemic anthrax cases are cutaneous and are contracted by close contact of abraded skin with products derived from infected herbivores, principally cattle, sheep, and goats. Such products might include hides, hair, wool, bone, and meal. Cutaneous anthrax is readily recognizable, presents a limited differential diagnosis, is amenable to therapy with any number of antibiotics, and is rarely fatal. While common in parts of Asia and sub-Saharan Africa, cutaneous anthrax is very rare in the United States; the last case was reported in 1992 (12). Inhalational anthrax, also known as woolsorters' disease, has been an occupational hazard of slaughterhouse and textile workers; immunization of such workers has all but eliminated this hazard in Western nations. As a weapon, however, anthrax would likely be delivered by aerosol and, consequently, be acquired by inhalation. A third type of anthrax, acquired through the gastrointestinal route (e.g., consuming contaminated meat) is exceedingly rare but was initially offered by Soviet scientists as an explanation for the Sverdlovsk outbreak.

Inhalational anthrax begins after exposure to the necessary inoculum, with the uptake of spores by pulmonary macrophages. These macrophages carry the spores to tracheobronchial or mediastinal lymph nodes. Here, *B. anthracis* finds a favorable milieu for growth and is induced to vegetate. The organism begins to produce an antiphagocytic capsule and at least three proteins, which appear to play a major role in virulence. These proteins are known as edema factor (EF), lethal factor (LF), and protective antigen (PA). Following the A-B model of toxicity (13), PA serves as a necessary carrier molecule for EF and LF and permits penetration into cells. Edema toxin results from the combination of EF + PA, lethal toxin results from the combination of LF + PA. These toxins result in necrosis of the lymphatic tissue, which in turn causes the release of large numbers of *B. anthracis.* The organisms gain access to the circulation, and an overwhelming fatal septicemia rapidly ensues. At autopsy, widespread hemorrhage and necrosis involving multiple organs is seen.

Inhalational anthrax generally occurs after an incubation period of 1 to 6 days (14). During the Sverdlovsk outbreak, however, spontaneous cases appeared to arise as late as 43 days after the assumed release date (7). Such late cases are unexplained but have potentially serious implications for postexposure management of victims of aerosol exposure. After the incubation period, a nonspecific flulike illness ensues, characterized by fever, myalgia, headache, a nonproductive cough, and mild chest discomfort. A brief intervening period of improvement sometimes follows 1 to 3 days of these prodromal symptoms, but rapid deterioration follows; this second phase is marked by high fever, dyspnea, stridor, cyanosis, and shock. In many cases, chest wall edema and hemorrhagic meningitis (present in up to 50% of cases [15]) may be seen late in the course of disease. Chest radiographs may show pleural effusions and a widened mediastinum, although true pneumonitis is not typically present. Blood smears in the later stages of illness may contain the characteristic gram-positive spore-forming bacilli. Death is universal in untreated cases and may occur in as many as 95% of treated cases if therapy is begun more than 48 hours after the onset of symptoms.

While early recognition of anthrax is likely to require a heightened degree of suspicion, the diagnosis is supported by gram-positive bacilli in skin biopsy material (in the case of cutaneous disease) or in blood smears. A preponderance of gram-positive bacilli in swabs of the nares or in appropriate environmental samples might support a diagnosis of anthrax where intentional release is suspected. Chest radiographs exhibiting a widened mediastinum in the proper setting of fever and constitutional signs and in the absence of another obvious explanation (such as blunt trauma, deceleration injury, or postsurgical infection) should also lead to a diagnosis of anthrax. This finding is only likely to occur late in the course of disease. Confirmation is obtained by culturing *B. anthracis* from blood.

### Disease Management

While endemic strains of *B. anthracis* are typically sensitive to various antibiotics, including penicillin G, antibiotic-resistant strains do (on rare occasion) occur naturally (16) and can be readily isolated in laboratories. For this reason, as well as the convenience of twice-daily dosing, many experts consider ciprofloxacin (400 mg intravenously (i.v.) q 12 h) the drug of choice for treating victims of terrorism or warfare. Doxycycline (100 mg i.v. q 12 h) is an acceptable alternative, although rare doxycycline-resistant strains of *B. anthracis* are known. Conversely, however, the much lower cost of tetracyclines compared to quinolones may factor into therapeutic decisions, especially where large numbers of patients are involved. These recommendations are based solely on in vitro data and data from animal models (17); no human clinical experience with these regimens exists. In cases of endemic anthrax, or where organisms are known to be susceptible, penicillin G (2 million units i.v. q 2 h or 4 million units i.v. q 4 h) is recommended.

Postexposure prophylaxis against anthrax may be achieved with oral ciprofloxacin (500 mg orally q 12 h) or doxycycline (100 mg orally q 12 h), and all persons exposed to a bioterrorist incident involving anthrax should be administered one of these regimens at the earliest possible opportunity. In cases of threatened or suspected release of anthrax, chemoprophylaxis can be delayed 24 to 48 hours, until the threat is verified. Chemoprophylaxis can be discontinued if the threat is found to be false. Levofloxacin and ofloxacin would be acceptable alternatives to ciprofloxacin. In addition to receiving chemoprophylaxis, exposed persons should be immunized. On the basis of animal data (wherein an appreciable number of unvaccinated primates died when antibiotics were withdrawn after 30 days of therapy) (18), chemoprophylaxis is best continued until the exposed persons has received at least three doses of vaccine (thus, for a minimum of 4 weeks). If vaccine is unavailable, some recommend that chemoprophylaxis be continued for 8 weeks (19). The available vaccine was licensed (for preexposure prophylaxis) by the U.S. Food and Drug Administration in 1970 and is prepared from a formalin-treated culture supernatent of an avirulent *B. anthracis* strain. It is given in a preexposure regimen at 0, 2, and 4 weeks, and at 6, 12, and 18 months. Persons at continuing risk for exposure should receive yearly boosters. Exposed persons should receive at least three doses (at 0, 2, and 4 weeks), assuming no further exposure is likely, before discontinuing chemoprohylaxis.

Recently, a number of hoaxes involving a threatened release of anthrax have been promulgated (19,20), and guidelines have now been published to assist in the management of such threats (19). When evaluating a threatened release of anthrax, the lack of volatility of the disease, as well as its inability to penetrate intact skin, should be taken into account. These factors make it unlikely, in most cases, that persons coming in contact with letters, packages, and other devices purported to contain anthrax will be at risk for aerosol exposure. Moreover, because energy is required to aerosolize anthrax spores, opening a letter, even if it contained anthrax, would be unlikely to place a person at substantial risk. For these reasons, postexposure prophylaxis may not be necessary in many cases of threatened anthrax dissemination.

Anthrax has little potential for person-to-person transmission; standard precautions are thus adequate for health-care workers treating anthrax patients. Anthrax, as well as other bacteriologic and viral weapons, has an incubation period of >24 hours. This characteristic is not shared by conventional, chemical, and nuclear weapons and makes decontamination of infected persons admitted to hospitals days after exposure unnecessary in most cases. However, in certain cases, such as exposure to a threat letter involving an unidentified substance, where anthrax cannot readily be ruled out by Gram stain or other rapid diagnostic procedures, decontamination may be warranted. In such cases, decontamination may be accomplished by removing clothing, sealing it in a plastic bag, and showering with copious amounts of soap and water. Environmental surfaces and personal effects may be treated with 0.5% hypochlorite after the area in which the agent was released is investigated (19).

In summary, even though anthrax may be among the most viable of biological weapons, it is also a weapon for which a licensed vaccine and good antimicrobial therapy and postexposure prophylaxis exist. Given the relatively short incubation period, and rapid progression of disease, however, identification of the exposed population within 24 to 48 hours and employment of therapeutic and prophylactic strategies are likely to present a challenge. Good intelligence regarding the capabilities of terrorist groups, as well as heightened awareness of the threat on the part of clinicians, first responders, and public health personnel remains a cornerstone of bioterrorism defense.

Dr. Cieslak is chief of Field Operations Department in the Division of Operational Medicine at the U.S. Army Medical Research Institute of Infectious Diseases at Ft Detrick, MD. Dr. Cieslak is working in the area of medical defense against biological warfare and terrorism.

Dr. Eitzen is chief of the Division of Operational Medicine at the U.S. Army Medical Research Institute of Infectious Diseases and adjunct associate professor of pediatrics and of military and emergency medicine at the Uniformed Services University of the Health Sciences in Bethesda, Maryland. He has worked in the area of medical defense against biological warfare and terrorism for the past 8 years.

### References

1. Exodus 9:1-12.
2. Pasteur L, Chamberlain C-E, Roux E. Compte rendu sommaire des experiences faites a Pouilly-le-Fort, pres Melun, sur la vaccination charbonneuse [French]. Comptes Rendus des seances De L'Academie des Sciences 1881;92:1378-83.
3. Turner M. Anthrax in humans in Zimbabwe. Cent Afr J Med 1980;26:160-1.
4. Harrison LH, Ezzell JW, Abshire TG, Kidd S, Kaufmann AF. Evaluation of serologic tests for diagnosis of anthrax after an outbreak of cutaneous anthrax in Paraguay. J Infect Dis 1989;160:706-10.
5. Report of a WHO group of consultants. Health aspects of chemical and biological weapons. Geneva: World Health Organization; 1970. p. 97-9.
6. Science Applications International Corporation. Effectiveness of medical intervention against battlefield levels of *Bacillus anthracis*. 1993.
7. Meselson M, Guillemin J, Hugh-Jones M, Langmuir A, Popova I, Shelokov A, Yampolskaya O, et al. The Sverdlovsk anthrax outbreak of 1979. Science 1994;266:1202-7.
8. Turell MJ, Knudson GB. Mechanical transmission of *Bacillus anthracis* by stable flies and mosquitoes. Infect Immun 1987;55:1859-61.
9. Titball RW, Turnbull PCB, Hutson RA. The monitoring and detection of *Bacillus anthracis* in the environment. Journal of Applied Bacteriology 1991; Suppl 70:9S-18.
10. Coker PR, Smith KL, Hugh-Jones ME. Anthrax in the USA. Proceedings of the Third International Conference on Anthrax, Plymouth, England, September 7-10, 1998:44 [abstract].
11. Sidell FR, Patrick WC, Dashiell TR, editors. Jane's chem-bio handbook. Alexandria (VA): Jane's Information Group; 1998. p. 229-44.
12. Centers for Disease Control and Prevention. Summary of notifiable diseases, United States, 1997. MMWR Morb Mortal Wkly Rep 1998;46:74.
13. Gill DM. Seven toxic peptides that cross cell membranes. In: Jeljaszewicz J, Walstrom T, editors. Bacterial toxins and cell membranes. New York: Academic Press; 1978. p. 291-332.
14. Brachman PS, Friedlander AM. Anthrax. In: Plotkin & Mortimer, editors. Vaccines. Philadelphia (PA): W.B. Saunders; 1994. p. 730.
15. Abramova FA, Grinberg LM, Yampolskaya OV, Walker DH. Pathology of inhalational anthrax in 42 cases from the Sverdlovsk outbreak of 1979. Proc Natl Acad Sci U S A 1993;90:2291-4.
16. Lightfoot NF, Scott RJD, Turnbull PCB. Antimicrobial susceptibility of *Bacillus anthracis.* Salisbury Medical Bulletin Suppl 1990;68:95-8.
17. Kelly DJ, Chulay JD, Mikesell P, Friedlander AM. Serum concentrations of penicillin, doxycycline, and ciprofloxacin during prolonged therapy in rhesus monkeys. J Infect Dis 1992;166:1184-7.
18. Friedlander AM, Welkos SL, Pitt MLM, Ezzell JW, Worsham PL, Rose KJ, et al. Postexposure prophylaxis against experimental inhalation anthrax. J Infect Dis 1993;167:1239-42.
19. Centers for Disease Control and Prevention. Bioterrorism alleging use of anthrax and interim guidelines for management-United States, 1998. MMWR Morb Mortal Wkly Rep 1999;48:69-74.
20. Sanchez R. California anthrax threats spawn costly wave of fear. Washington Post, January 11, 1999, section A, page 1.
